# Assessing antineoplastic drug contamination in South African oncology pharmacies: Workplace and biological monitoring

**DOI:** 10.1177/10781552251321150

**Published:** 2025-02-24

**Authors:** Ruan Botha, Gill Nelson, Paul Sessink, Derk Brouwer

**Affiliations:** 1 Occupational Health Division, School of Public Health, Faculty of Health Sciences, 37707 University of the Witwatersrand, Johannesburg, South Africa; 2 Department of Neurology, 115467 Barrow Neurological Institute, Phoenix, AZ, USA; 3 Exposure Control Sweden, Bohus-Björkö, Sweden

**Keywords:** Antineoplastic drugs, occupational exposure, oncology pharmacy, surface contamination, biological monitoring

## Abstract

**Introduction:**

Antineoplastic drugs (ADs) are hazardous substances commonly used in medical oncology, posing health risks to pharmacy personnel through occupational exposure. Despite safety guidelines, contamination of surfaces in oncology pharmacies remains prevalent. In this study, we assessed surface contamination and biological uptake of ADs in selected South African oncology pharmacies.

**Methods:**

Six pharmacies (three from each of two medical oncology service providers, SP1 and SP2) participated. Surface wipe samples were collected from three areas in each pharmacy (containment-primary engineering control (C-PEC), floor, adjacent surface) before and after decontamination procedures. Nine ADs were quantified, using high-pressure liquid chromatography tandem mass spectrometry (HPLC-MS/MS). 24-h urine samples were collected from six pharmacy personnel. The DeRmal Exposure Assessment Method (DREAM) was used to estimate exposure risks. The results were compared with those from an observational study that assessed compliance with safety protocols in the same six pharmacies.

**Results:**

Surface contamination was higher in SP1 than in SP2 pharmacies, with 5-fluorouracil (5-FU) being the most frequently detected AD. Post-decontamination samples showed a 17.4% reduction in 5-FU contamination, although SP1 pharmacies still had higher post-decontamination concentrations than SP2 pharmacies, especially on floors. Cyclophosphamide and ifosphamide were detected in the urine of two participants from SP1.

**Conclusion:**

We demonstrated higher contamination and occupational exposure risks in SP1 than in SP2 pharmacies, indicating a need for strict decontamination protocols and better use of personal protective equipment in the SP1 pharmacies. The SP2 pharmacies may serve as a model for oncology pharmacy safety in South Africa.

## Introduction

### Health effects of exposure to antineoplastic drugs

Much research has been conducted, describing exposure to antineoplastic drugs (ADs) in hospitals and medical oncology centres.^1–5^ Contamination is also common in oncology pharmacies worldwide.^6^ The genotoxic effects of ADs, such as sister-chromatid exchanges and chromosomal aberrations, were first described more than 40 years ago.^7,8^ Adverse health effects of AD exposure range from minor to severe and include tumours, leukaemia*,* congenital malformations, foetal loss, infertility, ectopic pregnancies, low birth weight, skin rashes, and other effects on body tissues with rapidly dividing cells (e.g., gastrointestinal tract, hair and nails).^4,9^ In response to the negative health effects associated with the handling of chemotherapy agents, the American Society of Health-System Pharmacists (ASHP) issued its first recommendations for oncology pharmacy staff on the safe handling of ADs in 1985.^10^ Many other guidelines regarding the safe handling of ADs have since been published.^11–14^

### Routes of exposure

Exposure can occur during drug preparation, handling, or cleaning activities. The most common route of exposure to ADs is through the skin due to direct contact or contact with contaminated surfaces. Thus, effective decontamination protocols, regular monitoring, and strict safety measures, including the use of personal protective equipment (PPE), are essential to protect those working with ADs.^15–17^ Exposure to ADs may also occur through inhalation if pharmacy staff are exposed to aerosols or dust particles containing ADs. Other routes of exposure include accidental ingestion and accidental injection during preparation.

Several studies have detected ADs in the urine of oncology pharmacy personnel.^18,19^ However, some researchers have found no evidence of AD uptake in oncology pharmacy personnel,^20–22^ which could be ascribed to good oncology pharmacy practice.

### Oncology pharmacy practices in South Africa

Parkin et al. estimated that the incidence of cancer would increase by 70% in sub-Saharan Africa from 2012 to 2030.^23^ The role of the oncology pharmacist in providing oncology care is thus more important than ever,^24^ including in emerging low- to middle-income countries (LMICs) like South Africa and other African countries where oncology pharmacy practice is evolving.^25^ Oncology pharmacy is not an officially recognised pharmacy speciality in South Africa; no registration as an oncology pharmacist is required, and no official or accredited training programmes are available.^26^ There are also no official oncology-specific guidelines in South Africa, and legislation about the preparation of ADs is outdated.^27^ Although many pharmacies where parenteral ADs are administered adhere to high standards,^28^ not all follow guidelines like the International Society of Oncology Pharmacy Practitioners (ISOPP) ‘Safe Handling’ of Cytostatics, and the European Society of Oncology Pharmacy (ESOP) Quality Standard for the Oncology Pharmacy Service (QuaPos), which include using PPE and conducting environmental and biological monitoring.^11,12^ The risk of exposure of oncology pharmacists and others who mix ADs thus remains a concern.

The objective of this study was to assess the potential for exposure of oncology pharmacy personnel to ADs based on contamination of pharmacy workspace surfaces.

## Methods

### Participating sites

Four service providers (SPs), representing four oncology pharmacy chains in the private sector in South Africa were approached to participate in the study; two refused without providing reasons. We successfully recruited six pharmacies located in urban areas: three in Gauteng province (SP1) and three in the Western Cape province (SP2).

### Antineoplastic drugs measured

The concentrations of nine ADs were measured on surfaces in the six pharmacies and in the urine of six pharmacy personnel (one from each pharmacy), viz. ifosphamide (IF), cyclophosphamide (CP), docetaxel (DOC), paclitaxel (PAC), gemcitabine (GEM), and 5-fluorouracil (5-FU), methotrexate (MTX), etoposide (ETO) and doxorubicin (DOX). These are some of the most commonly ADs used in South African medical oncology treatment protocols.

### Workplace surface wipe sampling

Using wipes (described in detail below), ADs were measured on the surface inside the containment primary engineering control (C-PEC) – the device in which ADs are mixed, the floor in front of the unit, and a surface adjacent to the C-PEC (e.g., a desk or trolley), from March to September 2023. In this way, three measurements of ADs were taken from each of the three surfaces on a single day: one in the morning before the mixing of ADs began (pre-decontamination measurement), one after the surface decontamination (cleaning) (post-decontamination measurement), and the third at the end of the day (end-of-day measurement). Thus, nine surface wipes were collected from each pharmacy. All pharmacies used standardised methods for decontamination, viz. 70% ethanol and sterile gauze for cleaning the C-PEC and adjacent surfaces, and soap, chlorine and water for cleaning the floor with a mop.

The sample wipe kits - provided by Exposure Control Sweden (ECS) - contained gloves, tissues, diluent (formic acid, CH_2_O_2_ solution), labels, and containers for storage. Surfaces of 0.5 m^2^ were drip-wetted with 17 ml of formic acid (CH_2_O_2_) solution, after which two dry wipes were used to dry the surface in a zigzag (Z) pattern. The method for sample collection followed ECS's procedures.^29^ The wipes were stored in airtight containers, frozen at −20 °C for preservation, and transported to the ECS laboratory at Radboud University in the Netherlands for analysis of ADs.

### Biological monitoring

Twenty-four hour urine samples were collected from each of the six participants on the same day as the surface wipe sample collection. Study participants collected their own urine samples in 10 ml vacuum tubes (similar to those used for blood samples), using a measuring cup and transfer device. Each participant recorded the volume of urine passed. Using this method, it was not necessary to collect all urine passed in the 24-h period. Ten vacuum tubes were provided to each participant for the collection of a maximum of 10 urine samples, which were frozen at −20°C on-site at the pharmacy in a dedicated freezer, or at the participants’ homes, immediately after collection (in which case, the samples were collected the following day and stored at −20°C).

Each urine sample from each participant was analysed for ADs independently (samples from the same individual were not pooled). The concentrations of ADs were measured in each individual urine sample and the mean concentrations were calculated for each participant. The limits of detection (LOD) and limits of quantification (LOQ) for each AD are provided in Table 1.

**Table 1.** Limits of detection and quantification for nine antineoplastic drugs for surface and urine samples.

	Surface concentration ng/cm^2^		Urine concentration ng/ml	
AD	LOD	LOQ	LOD	LOQ
5-fluorouracil	0.007	0.020	-	-
Gemcitabine	0.002	0.002	-	-
Ifosphamide	0.002	0.004	0.003	0.01
Cyclophosphamide	0.002	0.004	0.003	0.01
Methotrexate	0.001	0.002	-	-
Docetaxel	0.002	0.004	-	-
Paclitaxel	0.002	0.004	-	-
Etoposide	0.013	0.040	-	-
Doxorubicin	0.001	0.020	-	-

AD: antineoplastic drug, LOD: limit of detection, LOQ: limit of quantification.

Source: Exposure Control Sweden (www.exposurecontrol.net).

The samples were analysed using high-pressure liquid chromatography with tandem mass-spectrometry (HPLC-MS/MS).

### The DeRmal Exposure Assessment Method

The DeRmal Exposure Assessment Method (DREAM) was used to estimate exposure risks. This is a structured observational method consisting of an inventory and an evaluation component. The inventory component is completed by an occupational health professional and involves a hierarchically structured questionnaire/list of observation items designed to assess determinants and routes of exposure. It is divided into six modules: company, department, agent, job, task, and exposure. Each response within the questionnaire is allocated a specific score. The results of the DREAM analysis are captured in a freely available Microsoft-Excel-based tool in the language, Visual Basic for Applications.^30^ In the evaluation component of the DREAM, these scores are processed through a series of algorithms to generate indices of potential exposure, i.e., the amount of contaminant landing on the outer layer of work clothing, and actual exposure, i.e., the amount of contaminant reaching the exposed skin, and dermal exposure in arbitrary units (AU) for nine different body parts and at the task level.^31^ This approach allows for the detailed characterisation of the agent, the probability and intensity potential of exposure, and the assessment of hygiene practices, including the use of personal protective equipment in the workplace.

### Observational study

A cross-sectional pilot study was conducted in the same six oncology pharmacies in 2023 to assess work and workplace practices, and the findings were published.^28^ In brief, a 43-item checklist developed from international best practice guidelines was used to evaluate compliance in seven categories: engineering controls, administrative controls, personal protective equipment (PPE), education and training, medical and workplace monitoring, cleaning procedures, and waste handling. Data were collected through direct observations, document reviews, and personnel interviews. Compliance was scored as full, partial, or non-compliant for each category; total scores were calculated for each pharmacy and service provider. These scores were compared with the DREAM analysis risk estimates (described above) and the proportion of wipe samples with 5-FU concentrations ≥ 1 ng/cm^2^. 5-FU was selected because it was the AD for which the concentrations were highest in most of the pharmacies, and for which the number of measurements above the LOD was highest.

### Data analysis and interpretation of the results

Surface contamination data, including the 90^th^ percentiles of the distributions, were displayed, using the traffic light model of Sessink et al.^29^ This provided a visual indication of the extent of contamination for the areas sampled (traffic light model). Red indicates high contamination (≥10 ng/cm^2^), green low contamination (<1 ng/cm^2^) and orange and yellow as medium-high (1.0-<10 ng/cm^2^) and medium-low contamination (0.1-<1.0 ng/cm^2^), respectively. This is per German, Dutch and American national substance reference values as an indication of surface contamination with ADs.^32,33^

Descriptive statistics are provided for pre-decontamination and end-of-day concentrations combined (paired) since the end-of-day sample can be considered to be a ‘pre-decontamination’ sample of the following day. An imputation of non-detect values was conducted, using the online tool, NDExpo (www.expostats.ca), which uses regression on order statistics (ROS). An independent t-test was used to test differences between the mean numbers of patients for whom AD treatments were prepared in each SP as the exact numbers of AD preparations were not available. The Wilcoxon signed rank test, using C-PEC and adjacent surface pre- and post-decontamination data, was applied to evaluate the reduction of decontamination and to check for statistical significance. Spearman's rank correlation analysis was used to assess the strength and direction of the relationship between the mean C-PEC and adjacent surface, 5-FU concentrations (pre-decontamination and end-of-day measurements), the DREAM estimates, the observational study scores, and the total number of samples with concentrations ≥ 1 ng/cm^2^ (six correlations). Jeffery's Amazing Statistics Programme (JASP) version 0.18.3 (Amsterdam, the Netherlands - https://jasp-stats.org/) was used for statistical analysis. A p-value of <0.05 was considered to indicate statistical significance.

### Ethics approval

Ethics approval for the collection of surface wipes and urine samples was obtained from the University of the Witwatersrand Human Research Ethics Committee (HREC); certificate no. M190312.

## Results

### Surface contamination of antineoplastic drugs

None of the samples was positive for ETO, and only one sample was positive for DOX; both ADs were therefore omitted from the statistical analyses. The pre- and post-decontamination sample concentrations, and the end-of-the-day concentrations are displayed as “traffic-light models” in Tables 2 and 3, respectively. One hundred and sixty-four of the 252 (65.1%) wipe samples (beginning and end-of-day) were positive for at least one AD, i.e., concentrations were above the LOD. The surface contamination in 10 (6.1%) of the wipe samples was classified as ‘high’, in 42 (25.6%) it was classified as ‘medium-high’, in 65 (39.6%) as ‘medium-low’, and in 135 (82.3%) as ‘low’ or below the LOD. The highest concentration was observed for 5-FU from the C-PEC surface in pharmacy 2 (SP1), at 83.9 ng/cm^2^. Overall, 5-FU was the most frequently detected AD (n = 29), followed by CP (n = 28). There were 41 non-detect samples from SP1 and 48 from SP2.

**Table 2.**
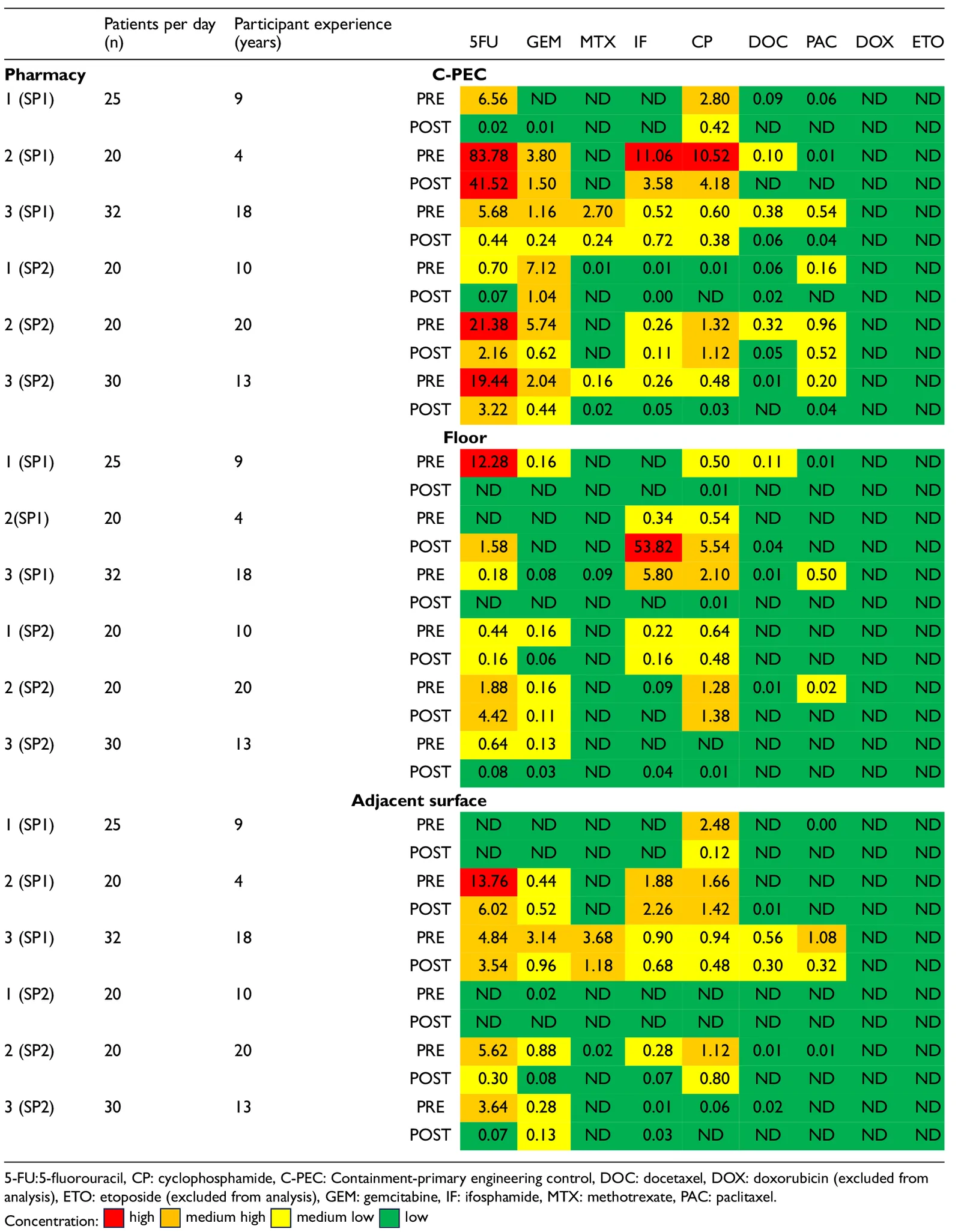
Traffic light model of surface concentrations of antineoplastic drugs per pharmacy, pre- and post-decontamination, including no. of patients per pharmacy per day (N = 252) and participant experience.

**Table 3.**
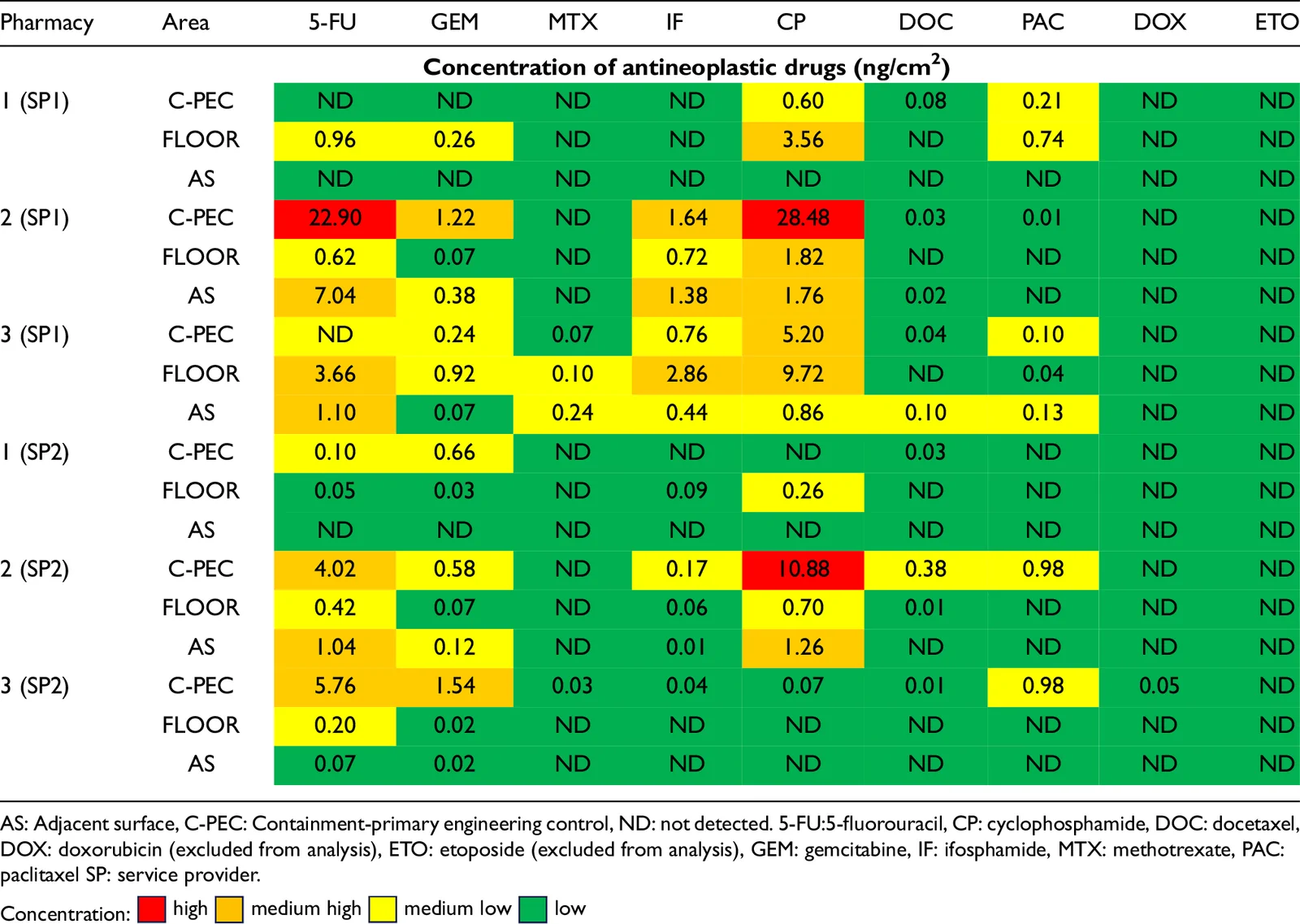
Traffic light model of end-of-day AD concentrations per pharmacy (N = 126).

### Comparison of antineoplastic drug concentrations between service providers

The summary statistics of the AD concentrations in the SP1 and SP2 pharmacies are shown in Table 4. There were notable discrepancies in surface concentrations between the two service providers. Pre- and end-of-day contamination concentrations were consistently higher in SP1 than in SP2 pharmacies, particularly for 5-FU. The proportions of AD concentrations in the highest contamination category (≥ 10 ng/cm^2^) were 2.8% and 1.2% in SP1 and SP2 respectively. Similarly, a higher proportion of samples in the SP1 than in the SP2 pharmacies fell into the “medium-high” concentration category (10.3% and 6.0%, respectively). The proportion of SP1 samples in the “medium-low” concentration category (13.5%) was also higher than that of SP2 samples (11.9%). There was a higher proportion of samples in the “low” concentration category (< 0.1 ng/cm^2^) in SP2 pharmacies (31.0%) than in SP1 pharmacies (22.8%). For the 90^th^ percentile, SP1, 5-FU and CP were high (≥ 10 ng/cm^2^) compared to SP2 which had none. In SP1, the 90^th^ percentile for MTX was low (< 0.1 ng/cm^2^). There was no significant difference between the mean number of patients processed in SP1 and SP2.

**Table 4.**
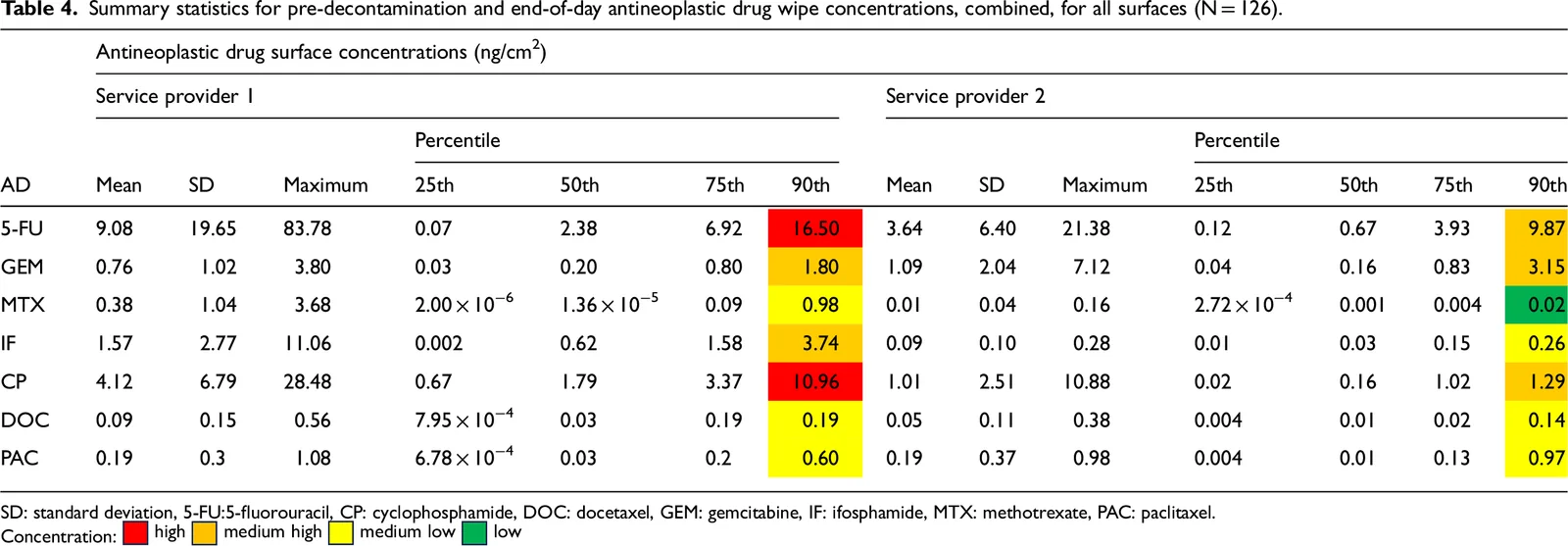
Summary statistics for pre-decontamination and end-of-day antineoplastic drug wipe concentrations, combined, for all surfaces (N = 126).

### Effectiveness of decontamination

The number of wipe samples that were positive for at least one AD decreased from 88 to 66 from the pre- to the post-decontamination sampling (17.4% reduction). Only two of the 126 post-contamination concentrations (1.6%) - one for 5-FU and one for CP (both in SP1 pharmacies) were classified as ‘high,’ compared to seven (5.8%) of the pre-decontamination concentrations. Sixteen measurements (12.7%) were in the ‘medium-high’ category - a 6.3% reduction in the number of samples in this concentration category; 24 measurements (19%) fell into the ‘medium-low’ category, 8.8% fewer than in the pre-decontamination period. The proportion of concentrations that was classified as ‘low’ increased by 19.1% (42 samples showed no detectable contamination). The differences between the pre- and post-decontamination mean concentrations were statistically significant for 5-FU (p = 0.002), GEM (p = 0.021), DOC (p = 0.024), and PAC (p = 0.021). None of the pharmacies had a cleaning protocol.

The 90^th^ percentiles and mean concentrations of most of the ADs were higher in the SP1 than the SP2 pharmacies (Table 4). The 90th percentile concentrations for 5-FU and CP in SP1 pharmacies were above the threshold of 10 ng/cm² (“high” concentration category), indicating a risk of exposure to these ADs for pharmacy personnel in the SP1 pharmacies. Specifically, the concentrations of 5-FU, MTX, IF, and CP were higher in the SP1 than the SP2 pharmacies. However, concentrations of GEM were slightly higher in SP2 than in SP1 pharmacies. Concentrations of DOC and PAC were similar in the pharmacies of both providers.

### Antineoplastic drugs detected in urine samples

Two of the six participants’ urine tested positive for IF and CP; both were from SP1 pharmacies. The mean concentrations in the first participant's urine were 3.77 ng/ml and 0.21 ng/ml for IF and CP, respectively. The mean concentrations in the second participant's urine were between the LOD and LOQ and could not be quantified.

### Comparison between DREAM estimates and observational study scores for 5-FU surface concentrations

The DREAM estimates and the scores calculated in the observational study are summarised in Table 5. Variations were observed in the task-weighted dermal exposure estimates across the six pharmacies. Pharmacy 1 and Pharmacy 2 (both SP1) exhibited the highest task-weighted potential dermal exposure estimates - both at medium-high levels of 38.3 AU. The estimate was slightly lower in pharmacy 3, at 37.4 AU. In contrast, service provider 2 pharmacies had significantly lower estimates (21.5 AU). This is reflected in the task-weighted actual dermal exposure estimates. SP2 pharmacies also scored higher than SP1 pharmacies in the work and work-related practices observational study (higher than 70% and lower than 50%, respectively).

**Table 5.** Comparison of DREAM estimates and observational study compliance scores.

Pharmacy	DREAM estimate (AU)		Observed
	Potential dermal exposure^a^	Actual dermal exposure^a^	Compliance score (%)
1 (SP1)	38.3	22.8	33.0
2 (SP1)	38.3	21.9	37.8
3 (SP1)	37.4	22.8	49.9
1 (SP2)	21.5	0.8	77.0
2 (SP2)	21.5	0.8	78.4
3 (SP2)	21.5	0.8	79.3

a task-weighted, AU: arbitrary units, DREAM: DeRmal Exposure Assessment Method, SP: service provider.

Table 6 shows the correlation between 5-FU surface concentrations and DREAM estimates for dermal exposure. The correlation was poor and not statistically significant (*ρ = *0.154, p = 0.770). The DREAM estimates were moderately but also non-significantly correlated with the number of surface wipe samples with concentrations > 1 ng/cm^2^. (*ρ = *0.344, p = 0.504). The observational study scores were significantly negatively correlated with the DREAM estimates (*ρ = −*0.926, p = 0.008). There was no correlation between the number of surface wipes with AD concentrations >1 ng/cm^2^ and the observational study scores (*ρ *= *−*0.087, p = 0.87).

**Table 6.** Correlation between 5-FU concentrations, DREAM estimates, observational study scores, and number of surface wipe samples with 5-FU concentrations ≥ 1 ng/cm^2^.

Variable		5-FU concentration (ng/cm^2^)	DREAM estimate^a^	No. wipe sample concentrations ≥ 1 ng/cm^2^
**5-FU concentration (ng/cm^2^)**	ρ	—		
	p-value	—		
**DREAM estimate** ^a^	*ρ*	0.154	—	
	p-value	0.770	—	
**No. surface wipe sample concentrations ≥1 ng/cm^2^** ^b^	*ρ*	0.899^a^	0.344	—
	p-value	0.015	0.504	—
**Observational study score**	*ρ*	0.143	−0.926	−0.087
	p-value	0.803	0.008	0.87

a for potential dermal exposure; ρ: Spearman's rank correlation coefficient; DREAM: DeRmal Exposure Assessment Method ^b^all antineoplastic drugs.

## Discussion

The ADs measured in this study were 5-FU, GEM, DOX, ETO, MTX, IF, CP, DOC and PAC. Initially, 5-FU, IF, CP and MTX were selected as these are some of the most commonly used ADs in patient treatment protocols. However, in addition to this, the laboratory also analysed the samples for GEM, DOX, ETO, DOC and PAC. The ADs most frequently detected in the analysis of the surface wipes were 5-FU, CP, and GEM. Previous studies in oncology pharmacies have also reported 5-FU, CP, and GEM to be among the most frequently detected ADs.^5,34^

The surface concentrations of 5-FU, CP, and IF were higher in the SP1 than in the SP2 pharmacies; GEM was the only AD with a lower mean concentration in SP1 than in SP2 pharmacies. In one of the SP1 pharmacies the pre-decontamination concentration of 5-FU on the C-PEC surface was 83.78 ng/cm^2^ – almost four times higher than the maximum concentration in any of the SP2 pharmacies (21.38 ng/cm^2^). Although this suggests that the contamination control measures used in the SP1 pharmacies were less effective than those used in the SP2 pharmacies, this is based on only one “high” 5-FU value.

Similar concentrations for 5-FU and CP to those measured in our study have been reported by others. Sottani et al. measured CP, 5-FU, GEM and platinum-containing ADs over a five-year period in Italy; 55% of samples were above the LOD.^35^ Chabut et al. measured nine ADs, including CP, 5-FU, GEM, IF, MTX and PAC in a longitudinal study in Canada.^36^ Surfaces were frequently contaminated with CP (34% of the positive samples were above the LOD) and GEM (16% were above the LOD). In a study by Sessink et al.^29^ in Belgium, the highest concentration recorded for 5-FU was 2.38 ng/cm^2^. The detection of 5-FU across all pharmacies in our study, even after decontamination (although at higher concentrations in SP1 pharmacies) points to a potentially higher risk of dermal exposure to this AD. Concentrations of other ADs such as CP were also more frequently higher in SP1 than in SP2 pharmacies on the three surfaces sampled.

Concentrations of ADs were also higher on adjacent surfaces in SP1 than in SP2 pharmacies, with 5-FU concentrations exceeding 13 ng/cm^2^ pre-decontamination in SP1, compared to a maximum of 5.62 ng/cm^2^ in SP2. The 90^th^ percentiles of 5-FU and CP were higher than 10 ng/cm^2^ in SP1 only, again suggesting that decontamination procedures were probably ineffective and work practices poor.

We did not detect a reduction in concentrations of all ADs across all surfaces after decontamination. Post-decontamination concentrations of 5-FU, IF, CP and DOC were higher than pre-decontamination concentrations in one of the SP1 pharmacies. One explanation for this could be the use of already contaminated cleaning equipment (mops).

Decontamination effectiveness will be more variable in real life workplace situations than in well-controlled studies as reported by Simon et al.^37^ and Palamini et al.^38^ In the latter study it was demonstrated that a sodium hypochlorite solution (NaOCl – 5.25%) removed 96.62% and 99.94% of IF and MTX, respectively on surfaces. They found that using 70% ethanol (EtOH), range 47.5–95.1% (mean 73.7%) was less effective. In a decontamination study by Hon et al. concentrations of ADs remained detectable in a controlled experiment, regardless of the cleaning agent used or the method of decontamination.^39^ Regardless, it is critical to thoroughly and appropriately decontaminate cleaning equipment daily.

The urine analysis results also indicated that contamination was higher in SP1 than in SP2 pharmacies. Only personnel from SP1 pharmacies had detectable concentrations of ADs (IF and CP) in their urine. Ndaw and Remy^40^ reported similar findings in a study in France, published in 2023: 53% of urine samples tested positive for CP, IF, MTX and α-fluoro-β-alanine (F-BAL - a metabolite of 5-FU) or any combination thereof. The positive urine results in SP1 personnel likely reflect inadequate PPE usage, as was documented in the observational study.^28^

Personnel in SP1 pharmacies used substandard PPE, such as plain latex gloves, single-use surgical masks, and woven, non-disposable gowns which did not cover the forearms. SP2 pharmacies were fully compliant in terms of the types of PPE used, including non-permeable, disposable gowns and other appropriate masks (such as FFP3 masks). As SP1 used substandard PPE, the gowning procedure was not correctly followed and forearms were exposed. Both SP1 and SP2 also did not comply entirely with donning and doffing of PPE as none had an ante-room in any of the pharmacies.

Although we did not measure dermal exposure, the DREAM allowed us to estimate this exposure, and supported our conclusion that working in an SP1 pharmacy poses a higher risk of dermal exposure than working in an SP2 pharmacy. The differences between the two SPs indicate variability in dermal exposure to ADs, which is likely due to differences in pharmacy practices, including PPE use. The task-weighted skin exposure estimates in two of the SP1 pharmacies were almost twice as high as those in the SP2 pharmacies, supporting the post-decontamination surface concentration findings.

The significant negative correlation between the DREAM estimates and the observational study scores indicates that poor implementation of, and adherence to safety protocols (specifically in SP1 pharmacies) potentially leads to higher dermal exposure levels.

### Limitations

The main limitation of this study was the small sample size. This was largely due to the unwillingness of some large service providers to participate in the study, and partly due to the high costs of transport and chemical analysis of the samples. Consequently, the findings cannot be generalized to all oncology pharmacies in South Africa. Moreover, it was impossible to estimate the quantities of the different ADs used in each pharmacy due to restrictions under the Protection of Personal Information (POPI) Act, and electronic prescription and invoice data could not be obtained.

## Conclusion

This is the first study to investigate workplace contamination and biological uptake of ADs in oncology pharmacies on the African continent. The study highlighted significant differences in surface contamination, decontamination effectiveness, and the potential for occupational exposure between the two medical oncology service providers. Concentrations of ADs were consistently higher in SP1 than SP2 pharmacies and the dermal exposure risks were higher, as evidenced by both surface concentrations and biological monitoring results. The findings suggest that decontamination protocols and PPE practices in SP1 pharmacies require improvement to reduce occupational exposure to ADs. Although the lower surface concentrations and absence of ADs in urine samples in SP2 pharmacy personnel suggest more effective implementation of safety protocols and better use of PPE, additional research is needed if the SP2 practices could serve as a model for improving oncology pharmacy practices in South Africa and protecting the health of oncology pharmacists.
